# The Influence of the Preservation Method and Gamma Irradiation Sterilization on TGF-*β* and bFGF Levels in Freeze-Dried Amnion Membrane (FD-AM) and Amnion Sponge

**DOI:** 10.1155/2021/6685225

**Published:** 2021-04-07

**Authors:** Heri Suroto, Deny M. Aryawan, Camilla A. Prakoeswa

**Affiliations:** ^1^Department of Orthopedics & Traumatology, Faculty of Medicine, Universitas Airlangga/Dr. Soetomo General Academic Hospital, Surabaya 60131, Indonesia; ^2^Faculty of Medicine, Universitas Airlangga, Surabaya 60131, Indonesia

## Abstract

**Background:**

Amnion grafts can be preserved as freeze-dried amnion membrane (FD-AM) and amnion sponge. Preserved grafts require to be sterilized by gamma irradiation. However, each step of the process could affect its biological properties. Even so, there are only a few studies that report the influence of the preservation method and gamma irradiation on growth factor levels in preserved amniotic grafts.

**Methods:**

This was an *in vitro* experimental study with a pretest-posttest group design using a consecutive sampling technique in one batch of amnion donors at a particular time. The amnion was made into FD-AM and amnion sponge preparations, and they were sterilized with gamma irradiation (15 kGy and 25 kGy). Nonirradiated specimens served as controls, and 20 mg of each specimen was pulverized to evaluate the growth factors levels using ELISA.

**Results:**

There were significant decreases in amnion sponge compared to the FD-AM, both in transforming growth factor beta (TGF-*β*) and basic fibroblast growth factor (bFGF) levels and in the preirradiated and 25 kGy postirradiated preparations (*p* ≤ 0.05). The growth factor levels in the preirradiated and postirradiated FD-AM (both 15 kGy and 25 kGy) showed significant differences (*p* ≤ 0.05). Likewise, the preirradiated amnion sponge group's growth factor levels compared with the postirradiated amnion sponge group also showed a significant decrease (*p* ≤ 0.05).

**Conclusion:**

TGF-*β* and bFGF levels were lower in amnion sponge than FD-AM. The FD-AM and amnion sponge preparations' growth factors levels were reduced following gamma irradiation sterilization. Although the decrease in growth factor levels is significant, the number of growth factor levels is still sufficient for tissue healing.

## 1. Introduction

The amnion membrane is a biomaterial that is widely known and used for various clinical applications in recent decades. The amnion membrane is derived from the placenta, an extraembryonic tissue, which consists of fetal components (chorion plate) and maternal components (decidua) [1]. The amnion membrane has specific properties, including anti-inflammatory, antibacterial, antiviral, antifibrotic, antiscarring properties and a very high tensile strength [2, 3]. It can also reduce the occurrence of brush tissue, protect wounds, reduce pain, increase the adhesion of basal epithelial cells, facilitate cell differentiation, and enhance re-epithelialization, making it ideal for tissue healing [3].

In vitro studies had proven that the amnion membrane produces growth factors that contribute to angiogenesis, re-epithelialization, and immunomodulation [4]. Using ELISA examination, a human amnion membrane is revealed to have seven growth factors: bFGF, TGF-*α*, TGF-*β*1, -*β*2, EGF, KGF, and HGF [5]. In wound healing, the Basic Fibroblast Growth Factor (bFGF) is responsible for granulation tissue formation, re-epithelialization, and tissue remodeling [6], while TGF-*β* inhibits ECM (extracellular matrix) degradation and increases collagen production [7].

There are some ways to preserve the amnion membrane; lyophilization or freeze-drying is one of the most commonly used methods [3]. Freeze-drying amnion membrane can maintain the amnion membrane viability for a long time without requiring a −80°C deep freezer for its storage [2]. Another method of amnion membrane preservation is by turning it into an amnion sponge. The amnion sponge is an amnion membrane minced to a size of about 250 *μ*m, mixed with adhesive material, and freeze-dried [1]. With its gelatinous form, the amnion sponge is doubtlessly easier to apply than the freeze-dried amnion membrane (FD-AM), which comes in a sheet-like form. The amnion sponge is a biomaterial that is easily obtainable, affordable, and easily applicable as an alternative material in accelerating the process of wound healing [1].

The sterilization technique is an integral part of the whole series of processes for making biomaterial preparations [3]. Sterilization is needed to prevent pathogen transmission or contamination. On the other hand, the chosen sterilization process must retain the biological potential of biomaterial preparation. Several ways have been formulated to sterilize the biomaterial, namely, using thermal, chemical, and electron radiation or gamma-ray sterilization [3]. The sterilization technique routinely used for amnion membrane sterilization at the Cell and Tissue Bank of Dr. Soetomo General Academic Hospital is 25 kGy gamma irradiation [8].

The commonly used method for radiation sterilization is by using gamma ray from Co-60 since it is practical, dependable, predictable, and has a high penetrability so the tissue can be sterilized even after its final packaging [3, 9]. As stated in ISO 11137 [10], sterilization of medical equipment can be carried out at a dose of 15 kGy or 25 kGy, depending on the product's initial bioburden, the number of a product's microorganism content [3]. Practically, the radiation dose usage varies from each tissue bank. Most tissue banks in the Asia Pacific have been using a dose of 25 kGy radiation sterilization. It is only recently that they have asked for a different dose since 25 kGy could negatively impact the grafts [11]. Most banks use 25 kGy as their reference radiation sterilization dose. On the other hand, some choose a higher dose to ensure the products' sterility, and some choose lower doses to preserve the tissue's biomechanical properties. Unfortunately, the use of radiation sterilization in high doses may induce chemical and physical changes that can alter the product's biological properties [12]. It is believed that the lower the initial number of bioburden, the smaller the dose of sterilization given and, thus, the smaller the biological damage caused [13].

To the best of our knowledge, only few studies report the effect of radiation sterilization on growth factor levels in amniotic allografts. There are also little data about the growth factor levels in amniotic grafts according to their preservation method. So, in this study, our goal is to determine the influence of gamma irradiation sterilization doses on growth factor levels between the different amnion membrane preservation methods, which were freeze-dried amnion membrane (FD-AM) and amnion sponge, by using the enzyme-linked immunosorbent assay (ELISA).

## 2. Materials and Methods

This study was an *in vitro* experimental study using the pretest-posttest group design. The sampling technique used in this study was consecutive sampling in one batch of amnion donors, consisting of eight patients, at a particular time who met the selection criteria. This study was performed in the Cell and Tissue Bank of Dr. Soetomo General Academic Hospital Surabaya and BATAN (National Nuclear Energy Agency of Indonesia), Jakarta, from June to December 2019.

The materials used in this study were eight amnion membranes from donors who met the eligibility criteria. Each placenta was divided into six specimens; three specimens for freeze-dried amnion membrane (FD-AM) and another three for amnion sponge preparations. According to its preservation method, different gamma radiation doses (15 kGy and 25 kGy) would then be given to one in each of the specimens. The remaining specimens that were not irradiated serve as a control. As much as 20 mg of each specimen was then collected and pulverized in the form of an amniotic powder to be evaluated for their growth factor levels using ELISA.

### 2.1. Making Freeze-Dried Amnion Membrane (FD-AM) and Amnion Sponge Preparations

The amnions were harvested from the fresh placentae of donors aged 18–42 years old who voluntarily donated their placenta and gave written consents. Donors were evaluated and confirmed to be free of substance abuse, human immunodeficiency viruses (HIV), hepatitis B viruses (HBV), hepatitis C viruses (HCV), and syphilis. Meconium-contaminated placentae and placentae from patients with pregnancy complications were excluded from this study.

The placentae were collected aseptically from the operating theatre in cesarean delivery or the delivery room in normal labor. The next step is evaluating the placentae for discoloration, the presence of debris or other contaminants, odor changes, and other signs of damage. If a placenta is in good condition, it is continued with the process of amnion and chorion separation. After being separated, the amnion was cleaned with normal saline (0.9% NaCl) to remove blood clots, mucus, and debris. It was then immersed in 0.9% NaCl solution in a sterile container, sealed, labeled, and ready to be delivered to the Cell and Tissue Bank of Dr. Soetomo General Academic Hospital inside a cool box at a temperature of −4°C.

The fresh amnion membranes that arrived at the Tissue Bank were evaluated for their container condition, sterility, and their administrative files' completeness. They were then put in a quarantine cupboard at −20°C until further processing.

The amnion membranes were processed using sterilized instruments in a clean room on a table with a sterile cloth. The amnion membranes were removed from the sterile containers and transferred to containers filled with 0.9% NaCl at room temperature. After the amnion membranes were at room temperature, approximately after 10 minutes, they were moved to the processing tray to be processed as amniotic grafts. Firstly, the amnion membranes were disinfected by soaking in 0.05% NaOCl solution for 10 minutes and then put in a water bath shaker (Julabo SW23) that had been filled with 0.9% NaCl in room temperature. The 0.9% NaCl needed to be changed ten times every 15 minutes. After that, the washed amnion membranes were stretched out and mounted on a sterile gauze with the chorion side facing the gauze.

For making freeze-dried amnion membranes (FD-AM), the amnion membranes were then deep-freezed at a temperature of −80°C for a minimum of 24 hours. The next step would be lyophilization (Lyophizer Lyovoc GT2) of the amnion membranes to a temperature of −40°C to −50°C that was carried out for 6–8 hours until the amnion membrane water content was 6-7%. The amnion membranes that had been lyophilized were then cut to the desired size and packed in three layers of polyethylene plastic sealed with a vacuum sealer. This process was carried out in a laminar airflow cabinet.

As for preparing the amnion sponge preparations, the same steps were also applied from the placenta tissue collection, amnion membranes disinfection with 0.05% NaOCl solution, and washing the amnion membranes with 0.9% NaCl until placing the stretched-out amnion on top of a sterile gauze. The difference for the amnion sponge was after those steps; the amnion was minced into small pieces and ground with normal saline with a ratio of 1 : 1 until the desired texture was achieved. The mixture was then molded as amnion sponge preparations and stored in a deep freezer at −80°C for a minimum of 24 hours before being lyophilized and, thus, packed in three layers of polyethylene plastic.

### 2.2. Sterilization

After vacuum-sealed in three polyethylene plastic layers, the freeze-dried amnion membrane (FD-AM) and amnion sponge preparations underwent sterilization. Sterilization was carried out by putting the FD-AM and amnion sponge preparations in their final packaging and irradiating them with 15 kGy and 25 kGy gamma-ray (Co-60) irradiation (Kimura, Chemical Plants Co., Ltd, Osaka, Japan). This process is executed in BATAN (National Nuclear Energy Agency of Indonesia), Jakarta.

### 2.3. Testing the Growth Factor Levels

As much as 20 mg of each of the preirradiated and irradiated FD-AM and amnion sponge preparations was collected and pulverized to check their growth factors levels. The pulverized FD-AM and amnion sponge were dissolved in 1 cc of 0.9% NaCl and then homogenized with a sonicator water bath (ELMA 1040 H) for 10 minutes. Furthermore, centrifugation was carried out at 1500 rpm for 5 minutes. The supernatant resulted from the centrifugation was then collected to measure the levels of bFGF and TGF-*β* using the ELISA technique (ELISA Kit BTLab human bFGF) [14].

### 2.4. Data Analysis

The data obtained were analyzed using a paired *t*-test to assess the growth factors' levels before and after radiation (both with 15 kGy and 25 kGy irradiation) and an independent *t*-test to compare the TGF-*β* and bFGF levels between the freeze-dried amnion membrane (FD-AM) and amnion sponge groups in normally distributed data.

## 3. Results

**Figure 1:**
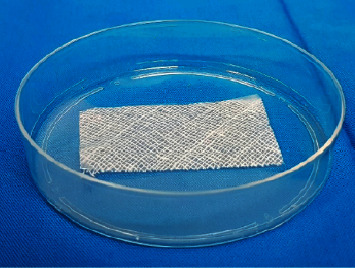
Freeze-Dried Amnion Membrane (FD-AM) in a Petri dish.

**Figure 2:**
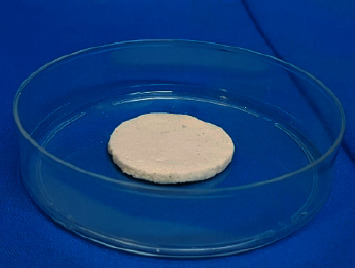
Amnion sponge in a Petri dish.

This research was conducted experimentally using a pretest-posttest group design. We have carried out initial measurements (pretest), followed by treatment and final measurements (posttest) in both groups. Our study evaluates the influence of both the gamma-ray irradiation and the method of preservation on the TGF-*β* and bFGF levels in amniotic grafts, namely, freeze-dried amnion membrane (FD-AM) and the amnion sponge. The FD-AM and amnion sponge preparations can be seen in Figures 1 and 2 below.

The initial procedure was performed by measuring the TGF-*β* and bFGF levels in preirradiated FD-AM and comparing them with the TGF-*β* and bFGF levels of the amnion membrane that had been further processed into an amnion sponge. After the FD-AM and amnion sponge underwent radiation sterilization with 15 kGy and 25 kGy doses of gamma ray, we evaluated both preparations' TGF-*β* and bFGF. We compared them with their preirradiated growth factor levels. We expected a decrease in the growth factor levels both after being processed into an amnion sponge and after being given gamma-ray radiation since those treatments could cause fragmentation of the amino acid that made up the growth factors.

The independent *t*-test result of the TGF-*β* and bFGF levels showed a significant decrease in the amnion sponge compared to the FD-AM, both in the preirradiated and 25 kGy postirradiated preparations. The TGF-*β* and bFGF levels in the preirradiated and postirradiated freeze-dried amnion membrane (FD-AM) compared with the amnion sponge are shown in Table 1.

The outcome of the paired-sample *t*-test of the TGF-*β* levels in the preirradiated amnion sponge group compared with the postirradiated amnion sponge groups, both 15 kGy and 25 kGy, showed a significant decrease (*p* < 0.0001) (Table 2). On the other hand, there was not a significant difference in the TGF-*β* level comparison in the preirradiated and postirradiated FD-AM both for 15 kGy and 25 kGy irradiation (*p*=0.012) (Table 3). We also calculated the percentage decrease in TGF-*β* levels of the 25 kGy postirradiated specimens from the preirradiated specimens in the FD-AM, as well as the amnion sponge. The mean percentage decrease in TGF-*β* levels was 82.35 ± 7.58% in FD-AM and 73.89 ± 2.61% in amnion sponge (Table 4).

The paired-sample *t*-test results of the bFGF levels of the preirradiated freeze-dried amnion membrane (FD-AM) compared to the 15 kGy and 25 kGy postirradiated FD-AM show significant differences (*p*=0.001 and *p* < 0.0001, respectively) (Table 5). Likewise, the difference was also statistically significant in bFGF levels comparisons of the preirradiated and postirradiated amnion sponge preparations, both with 15 kGy and 25 kGy (*p* < 0.0001) (Table 6). The mean percentage decrease of the bFGF levels in 25 kGy postirradiated specimens from the preirradiated specimens in the FD-AM was 30.57 ± 10.33%. The results of bFGF levels before and after radiation sterilization, as well as the percentage decrease in FD-AM and amnion sponge groups, can be seen in Table 7.

There were no significant differences in TGF- *β* and bFGF levels' mean percentage decrease between the FD-AM and the amnion sponge (*p*=0.01 and 0 = 0.662, respectively) from the independent *t*-test results (Table 8).

## 4. Discussion

### 4.1. Preservation Method

The amnion membrane contains collagen matrices and major bioactive molecules such as various growth factors. As we all know, the levels of growth factors in amnion membranes varied between individuals and can affect their biological effects [15]. At present, there are plenty of amnion membrane preservation methods available, namely, air-drying, freeze-drying, and glycerol preserving [16]. Those processes allow changes in the biological properties, affecting the growth factor levels in the amnion membrane's final form.

The results of the TGF-*β* and bFGF levels in the freeze-dried amnion membrane (FD-AM) preparations compared to the amnion sponge preparations in this study showed statistically significant differences in which the levels are lower in the amnion sponge preparations (*p* < 0.05). It may be due to the fact that the amnion sponge underwent a different step, in which it was minced and ground with 0.9% NaCl to create a different texture, whereas the FD-AM does not go through a minced state. It shall be considered that the actual TGF-*β* and bFGF levels are higher than those in the results of this study since both the FD-AM and the amnion sponge need to be pulverized to evaluate the growth factor level using the ELISA technique.

The amnion membrane goes through various stages from processing, storage, and preparation before being used as an amniotic graft. Each of these stages could influence the amniotic graft's growth factor levels [17]. Previous studies have shown that methods such as freeze-drying preservation and radiation sterilization significantly impact the amniotic allograft histological and biophysical properties [12].

According to Ihsan [18], decreased EGF levels in freeze-dried amnion membranes occur due to several things, such as an injury during freezing, freeze-drying, packing, and radiation. He explained that the EGF levels in fresh and freeze-dried amnion membranes in his research were lower than other researchers' results. It can happen due to the different extraction processes and ELISA tests used. The reduction in EGF levels in this study can occur due to several stages of the process involved during the manufacture of freeze-dried amnion membranes. Some tissue damage may occur during the freezing of the sample, the process of freeze-drying, and radiation [18].

The next step is the freeze-drying process; drying the tissue was performed directly from the ice phase without going through the liquid phase to prevent tissue autolysis. Changes in temperature during freeze-drying are thought to cause sample damage. The final stage of processing the freeze-dried amnion membrane is radiation sterilization. Interaction of radiation with material occurs in two ways, namely, directly due to radiation ionization itself and indirect ways through the formation of free radicals. The radiation process is carried out at low temperatures, free of water, and free of oxygen (vacuum) to prevent the effects of oxygen radicals from radiation [18].

Amnion membrane freezing aims to prepare the freeze-drying process and to reduce tissue antigen properties. Freezing at −80°C can cause freezing injuries in the form of tiny ice crystals or changes in the concentration and composition of fluids in cells. It disrupts the diffusion and osmosis process of the cells resulting in cell damage. In addition, freezing also causes protein stress, which results in protein denaturation. The longer the freezing process occurs, the more the ice crystals formed, so the cell damage becomes more severe. In this study, the freezing of the amnion membrane lasted 24–36 hours [18]. On the other hand, the amnion sponge is an amnion membrane that is crushed to a size of about 250 *μ*m mixed with an adhesive material and processed by the freeze-drying method at room temperature [19]. This study expected a significant decrease in the amnion sponge's growth factor levels compared to the freeze-dried amnion membrane since the amnion membrane requires being ground before processed as an amnion sponge.

Russo et al. used the “amnion membrane powder” manufacturing stage, similar to this study [17]. The preirradiation bFGF levels obtained in the study were 21.72 ± 5.33 pg for each gram of fresh amnion membrane used, while TGF-*β*1 5.37 ± 0.93 pg/g [17]. It was difficult to compare Russo et al.'s study results directly with this study because they refer to the amniotic wet weight as a comparison. Hence, the units that are used are different. In this study, each amnion membrane donor's level of growth factor varies [17]. Wu et al. made extracts from five fresh amnion membranes without lyophilization and gamma sterilization for their study and obtained higher bFGF levels of 646.4 pg/ml (172–1913 pg/ml) [20]. However, TGF-*β* levels in the study were much lower; one of the TGF-*β* isoforms, namely, TGF-*β*1, was only 346 pg/ml (257–468 pg/ml) [14]. It is similar to the study conducted by López-Valladares et al. that showed the total protein content. bFGF, HGF, KGF, and TGF-*β*1 for each amniotic sample varied and correlated with gestational age and donor age [21].

A freeze-dried amnion membrane can be stored and remain stable in various storage conditions without losing its clinical function. Depending on the usage and storage, a cryopreserved amnion membrane can be used after a year, while a freeze-dried and irradiated amnion membrane can last for several years [22]. Freeze-drying means removing the water from the amnion membrane by sublimation, thus inhibiting the chemical reactions that could cause tissue destruction [23]. There were doubts whether the diminishing growth factor levels in the preserved and irradiated amnion membrane could make wound healing ineffective. However, a study [24] found an insignificant difference between a cryopreserved amnion membrane and a lyophilized amnion membrane in corneal surface reconstruction. Moreover, previous literature reported that, in an animal model study, a lyophilized amnion membrane is as potent as a nonlyophilized amnion membrane when used as a biomaterial for corneal reconstruction [2]. Those results proved that the reduced growth factor levels in the freeze-dried amnion membrane are still acceptable for clinical use.

### 4.2. Sterilization Method

Sterilization is an essential process in making biomaterials, but most sterilization techniques can affect the product's biological properties. However, radiation sterilization by using gamma ray is cold sterilization. It is recommended for use in biological tissue due to its ability to inactivate microorganisms without a significant rise in temperature of the irradiated material. Moreover, gamma irradiation also has a high penetrability so that the tissue can be sterilized even if it is in bulk after the final packaging [9].

As stated in ISO (the International Organization for Standardization) 11137, sterilization of medical equipment can be carried out at a dose of 15 kGy or 25 kGy according to its bioburden [10]. It is thought that the lower the initial number of bioburden, the smaller the dose of sterilization given and the smaller the biological damage caused [13]. It needs to be considered that the selected doses for radiation sterilization must be reliably effective enough to destroy microorganisms and cause as minimal damage as possible [3].

This study showed a significant difference in TGF-*β* and bFGF levels in preirradiated and postirradiated products, both in the freeze-dried amnion membrane (FD-AM) and the amnion sponge. After calculating TGF-*β* and bFGF levels' reduction in both preparations, the most percentage decrease was found in the TGF-*β* level of the 25 kGy postirradiated freeze-dried amnion membrane (FD-AM) with the number of 82.35 ± 7.58%. The slightest percentage decrease was in the bFGF levels of the 25 kGy postirradiated freeze-dried amnion membrane (FD-AM), which decreased to 30.57 ± 10.33% from the preirradiated preparations.

Gamma irradiation with Co-60 for graft sterilization could impact the tissue's biomechanical properties in a dose-dependent manner. Unfortunately, tissue banks have to ensure that the grafts are irradiated with a dose high enough to have a sterility assurance level (SAL) of 10^−6^ while preserving their function as an in vivo biomaterial [25]. Radiation sterilization can cause the degradation of growth factors that play a role in the process of osteoinduction (bone morphogenetic protein and TGF-*β*) [26]. A study conducted by Costa et al. in 2006 found the bFGF level of the postirradiated amnion membrane powder was 425.7 ± 153.9 pg/ml, while a level of around 500 pg/ml or 0.5 ng/ml is needed to stimulate endotenon and epitenon proliferation [27]. The smallest amount of bFGF found in our study was 15.50 ng/mL from the 25 kGy postirradiated amnion sponge. From previous literature, it is proven that although the graft had been preserved as an amnion sponge and given 25 kGy irradiation, it was still adequate for endotenon and epitenon proliferation [27].

Russo et al. measured the levels of the epithelial growth factor (EGF) of the 25 kGy radiation amnion preirradiated and postirradiated membrane powder from two donors [17]. After examining the two samples, it was found that the EGF concentration of the postirradiated amnion membrane only slightly decreased to 98.72 ± 0.67% from the preirradiated level [17]. Skopiński et al. showed that the amnion membrane treated with gamma sterilization at a dose of 35 kGy had a different effect from the nonirradiated amnion membrane on endothelial cell culture [28]. Russo et al. measured Epithelial Growth Factor (EGF) levels before and after 25 kGy gamma irradiation in the amnion membrane powder from two donors [17]. From the examination of the two samples, it was found that the postirradiation EGF concentration had only slightly decreased to 98.72 ± 0.67% from the preirradiation level [17].

Ionizing radiation at a particular dose can cause damage to cellular structures. On the other hand, the cells have a complicated repair system to defend themselves from radiation damage. This system's efficiency, especially cells' survival, depends on the genes encoded by the DNA sequences. Thus, the status of genes and gene products' activity rely on DNA sequences' integrity [29, 30]. Radiation exposure leads to cross-link reaction and protein fragmentation, aggregation, and oxidation [24]. Endothelial cells' death by apoptosis or mitotic death is caused by radiation due to the changes in the endothelial cells' function. It can cause a decrease in the quantity and quality of various growth factors or other elements in a network directly and indirectly. Determining the radiation dose is very important to maintain the quantity and quality of various growth factors in allografts such as the amnion membrane [31].

The most remarkable tissue graft changes after radiation sterilization would be the loss of the graft's mechanical integrity and its biological substances, including the growth factors. Nonetheless, the graft still has other favorable properties, such as its barrier functions that can prevent wound contamination, thus validating its use when there is an indication of open wound coverage [32]. A study reported no significant changes in amnion membranes' structure after doses of 15, 20, 25, and 30 kGy gamma irradiation [33].

Even though the growth factor levels will likely decrease after given radiation sterilization, some studies say giving the grafts a dose of 25 kGy radiation sterilization still maintains their function to provide tissue healing. A study conducted by Djefal et al. concluded that maintaining a dose of 25 kGy of gamma irradiation could be substantiated to sterilize human freeze-dried amnion membranes (FD-AM) [34]. Yusof and Hilmy stated that a dose of 25 kGy radiation could be easily given to amnions with low bioburden to achieve sterility assurance level 10^−6^ [3]. In comparison, Deocaris et al. recommended a dose of 25 and 35 kGy irradiation for amnion membrane sterilization to balance the requirement for product sterility as well as to prevent excessive destruction to the graft [24]. However, although tissue sterilization is an indispensable process in making amnion grafts, it needs to be remembered that tissue sterilization is by no means a replacement for proper and hygienic tissue handling.

## 5. Conclusions

Preserved amniotic graft preparations, including freeze-dried amnion membrane (FD-AM) and amnion sponge, have lower growth factor levels than the fresh amnion grafts. This study found that the TGF-*β* and bFGF levels were lower in the amnion sponge than in the FD-AM. The lessening of growth factors in preserved amniotic is doubtlessly inevitable. However, it needs to be considered that a statistically significant decrease in growth factor level does not necessarily mean the grafts are unfit for clinical use. Although preserved amniotic grafts have lower growth factor levels, studies proved that the preserved amnions are as potent as a nonpreserved amnion membrane in clinical applications.

Both FD-AM and amnion sponge preparations showed a reduction of growth factor level after given gamma irradiation sterilization with doses of 15 kGy and 25 kGy. Gamma irradiation sterilization is an essential factor in processing amniotic grafts that cannot be compromised. It is suggested that graft sterilization is carried out with a dose of 25 kGy gamma irradiation to assure a sterility assurance level (SAL) of 10–6 is reached.

This study used FD-AM and amnion sponge and evaluated their TGF-*β* and bFGF levels. We hope there could be more research on the effect of radiation sterilization in other types of amniotic grafts, as well as an in vivo study to analyze the difference of growth factor level on living tissue.

## Figures and Tables

**Table 1:** Comparison of TGF-*β* and bFGF levels in the preirradiated and postirradiated freeze-dried amnion membrane (FD-AM) with the amnion sponge.

	TGF-*β*			bFGF		
	FD-AM	Amnion sponge	*p* value	FD-AM	Amnion sponge	*p* value
Preirradiated (ng/L)	2733.5 ± 589.9	1205 ± 173.1	0.002^ab^	38.26 ± 6.5	27.54 ± 2.7	0.001^ab^
Postirradiated 15 kGy (ng/L)	597.5 ± 86.6	455.8 ± 104.6	0.011^ab^	29.56 ± 5.9	22.49 ± 3.1	0.009^ab^
Postirradiated 25 kGy (ng/L)	448.6 ± 108.1	314 ± 53.4	0.004^ab^	26.42 ± 5.2	18.54 ± 2.1	0.001^ab^

^a^ *p* value<0.05. ^b^Independent *t*-test.

**Table 2:** Comparison of TGF-*β* levels in amnion sponge before and after radiation sterilization.

	Postirradiated 15 kGy	Postirradiated 25 kGy
Preirradiated	<0.0001^ab^	<0.0001^ab^

^a^ *p* value<0.05. ^b^Paired-sample *t*-test.

**Table 3:** Comparison of TGF-*β* levels in the freeze-dried amnion membrane (FD-AM) before and after radiation sterilization.

	Postirradiated 15 kGy	Postirradiated 25 kGy
Preirradiated	0.012^ab^	0.012^ab^

^a^ *p* value<0.05. ^b^Paired-sample *t*-test.

**Table 4:** TGF-*β* levels in the freeze-dried amnion membrane (FD-AM) and amnion sponge before and after radiation sterilization.

Donor	Freeze-dried amnion membrane (FD-AM)				Amnion sponge			
	Preirradiated (ng/L)	Postirradiated 15 kGy (ng/L)	Postirradiated 25 kGy (ng/L)	Δ preirradiated postirradiated 25 kGy (%)	Preirradiated (ng/L)	Postirradiated 15 kGy (ng/L)	Postirradiated 25 kGy (ng/L)	Δ preirradiated postirradiated 25 kGy (%)
A	1316	465	451	65.7	870	532	252	71.0
B	2592	503	395	84.7	1030	324	241	76.6
C	2967	562	452	84.8	1200	360	294	75.5
D	2980	590	420	85.9	1230	390	310	74.8
E	2989	610	343	88.5	1270	410	340	73.2
F	2999	650	360	88.0	1320	450	370	72.0
G	3010	680	478	84.1	1340	560	395	70.5
H	3015	720	690	77.1	1380	620	310	77.5
Mean	2733.5 ± 589.9	597.5 ± 86.6	448.6 ± 108.1	82.35 ± 7.58	1205 ± 173.1	455.8 ± 104.6	314 ± 53.4	73.89 ± 2.61

**Table 5:** Comparison of bFGF levels in amnion sponge before and after radiation sterilization.

	Postirradiated 15 kGy	Postirradiated 25 kGy
Preirradiated	<0.0001^ab^	<0.0001^ab^

^a^ *p* value<0.05. ^b^Paired-sample *t*-test.

**Table 6:** bFGF levels in the freeze-dried amnion membrane (FD-AM) and amnion sponge before and after radiation sterilization.

Donor	Freeze-dried amnion membrane (FD-AM)				Amnion sponge			
	Preirradiated (ng/mL)	Postirradiated 15 kGy (ng/mL)	Postirradiated 25 kGy (ng/mL)	Δ preirradiated postirradiated 25 kGy (%)	Preirradiated (ng/mL)	Postirradiated 15 kGy (ng/mL)	Postirradiated 25 kGy (ng/mL)	Δ preirradiated postirradiated 25 kGy (%)
A	27.55	20.65	17.60	36.12	25.75	17.30	15.50	39.81
B	29.90	25.35	23.55	21.24	23.10	19.25	18.95	17.97
C	37.50	30.25	28.85	23.07	25.95	23.15	17.05	34.30
D	39.50	33.95	30.45	22.91	26.70	21.10	16.40	38.58
E	40.20	33.40	30.70	23.63	28.10	24.60	18.70	33.45
F	41.30	22.65	20.20	51.01	29.40	22.90	20.50	30.27
G	43.40	35.70	30.74	29.17	30.50	25.20	21.60	29.18
H	46.70	34.50	29.25	37.37	30.80	26.40	19.60	36.69
Mean	38.26 ± 6.5	29.56 ± 5.9	26.42 ± 5.2	30.57 ± 10.33	27.54 ± 2.7	22.49 ± 3.1	18.54 ± 2.1	32.53 ± 6.96

**Table 7:** Comparison of bFGF levels in the freeze-dried amnion membrane (FD-AM) before and after radiation sterilization.

	Postirradiated 15 kGy	Postirradiated 25 kGy
Preirradiated	<0.001^ab^	<0.0001^ab^

^a^ *p* value<0.05. ^b^Paired-sample *t*-test.

**Table 8:** Comparison of TGF-*β* and bFGF mean percentage decrease between the preirradiated and postirradiated 25 kGy freeze-dried amnion membrane (FD-AM) and amnion sponge.

		FD-AM	Amnion sponge
		bFGF Δ preirradiated postirradiated 25 kGy	TGF-*β* Δ preirradiated postirradiated 25 kGy
FD-AM	TGF-*β* Δ preirradiated postirradiated 25 kGy	—	0.01^ab^
Amnion sponge	bFGF Δ preirradiated postirradiated 25 kGy	0.662^b^	—

^a^ *p* value<0.05. ^b^Independent *t*-test.

## Data Availability

The data used to support the findings of this study are included within the article.

## References

[1] Munadziroh E., Yuliati A., Ragillia A. N. (2018). Cytotoxicity test of sponge amnion on BHK-21 fibroblast cell. *Journal of International Dental and Medical Research*.

[2] Libera R. D., Melo G. B. d., Lima A. d. S., Haapalainen E. F., Cristovam P., Gomes J. Á. P. (2008). Assessment of the use of cryopreserved x freeze-dried amniotic membrane (AM) for reconstruction of ocular surface in rabbit model. *Arquivos Brasileiros de Oftalmologia*.

[3] Yusof N., Hilmy N. (2017). *Sterilization of Amnion. Human Amniotic Membrane*.

[4] Grzywocs Z., Pius-Sadowska E., Klos P. (2014). Growth factors and their receptors derived from human amniotic cells in vitro. *Folia Histochemica et Cytobiologica*.

[5] Koizumi N., Inatomi T., Sotozono C., Fullwood N. J., Quantock A. J., Kinoshita S. (2000). Growth factor mRNA and protein in preserved human amniotic membrane. *Current Eye Research*.

[6] Barrientos S., Stojadinovic O., Golinko M. S., Brem H., Tomic-Canic M. (2008). Perspective article: growth factors and cytokines in wound healing. *Wound Repair and Regeneration*.

[7] Litwiniuk M., Grzela T. (2014). Amniotic membrane: new concepts for an old dressing. *Wound Repair and Regeneration*.

[8] Ferdiansyah S., Noer T. W., Martanto S., Rizaliyana T. H. (2005). *Buku Pedoman Kerja Bank Jaringan [Tissue Bank Technical Guidebook]*.

[9] Chou J. W., Skornicki M., Cohen J. T. (2018). Unintended consequences of the potential phase-out of gamma irradiation. *F1000Research*.

[10] International Organization for Standardization (2017). *Sterilization of Health Care Products — Radiation — Part 2: Establishing the Sterilization Dose*.

[11] Yusof N. (2007). *“Radiation Sterilization Dose Establishment for Tissue Grafts – Dose Setting and Dose Validation*.

[12] Singh R., Singh D., Singh A. (2016). Radiation sterilization of tissue allografts: a review. *World Journal of Radiology*.

[13] Suryani N. (2013). Comparation of drying method on the amnion resorption in the SBF solution. *Beta Gamma*.

[14] Sankar W. N., Vanderhave K. L., Matheney T., Herrera-Soto J. A., Karlen J. W. (2013). The modified dunn procedure for unstable slipped capital femoral epiphysis. *Journal of Bone and Joint Surgery*.

[15] Choi S., Reddy P., Joo C. K. (2011). HDAC inhibition and graft versus host disease. *Molecular Medicine*.

[16] Hennerbichler S., Reichl B., Pleiner D., Gabriel C., Eibl J., Redl H. (2007). The influence of various storage conditions on cell viability in amniotic membrane. *Cell and Tissue Banking*.

[17] Russo A., Bonci P., Bonci P. (2012). The effects of different preservation processes on the total protein and growth factor content in a new biological product developed from human amniotic membrane. *Cell and Tissue Banking*.

[18] Ihsan P. (2009). The difference of epidermal growth factor concentration between fresh and freeze-dried amniotic membranes. *Journal of Oftamologia Indonesia*.

[19] Keliat I. (2017). *Aplikasi Sponge Amnion Dengan Ukuran Partikel Serbuk Berbeda Pada Luka Pasca Pencabutan Gigi Tikus Wistar Terhadap Ekspresi VEGF, Jumlah Pembuluh Darah Baru, Dan Jumlah Kolagen [Amnion Sponge Application with Different Powder Particles Size in Post-extraction Wounds of Wistar Rats on VEGF Expression, New Blood Vessels Formation, and Collagen Amount]*.

[20] Wu M.-F., Stachon T., Langenbucher A., Seitz B., Szentmáry N. (2016). Effect of Amniotic membrane suspension (AMS) and amniotic membrane homogenate (AMH) on human corneal epithelial cell viability, migration and proliferation in vitro. *Current Eye Research*.

[21] López-Valladares M. J., Rodríguez-Ares M. T., Touriño R., Gude F., Teresa Silva M., Couceiro J. (2010). Donor age and gestational age influence on growth factor levels in human amniotic membrane. *Acta Ophthalmologica*.

[22] Dekaris I., Gabri&cacute N. (2009). Preparation and preservation of amniotic membrane. *Eye Banking*.

[23] Riau A. K., Beuerman R. W., Lim L. S., Mehta J. S. (2010). Preservation, sterilization and de-epithelialization of human amniotic membrane for use in ocular surface reconstruction. *Biomaterials*.

[24] Deocaris C. C., Abad L. B., Enriquez E. P. (2005). Radiolytic damage to freeze-dried human amniotic membrane. *Philipipine Journal of Science*.

[25] McGilvray K. C., Santoni B. G., Turner A. S., Bogdansky S., Wheeler D. L., Puttlitz C. M. (2011). Effects of 60Co gamma radiation dose on initial structural biomechanical properties of ovine bone-patellar tendon-bone allografts. *Cell and Tissue Banking*.

[26] Ijiri S., Yamamuro T., Nakamura T., Kotani S., Notoya K. (1994). Effect of sterilization on bone morphogenetic protein. *Journal of Orthopaedic Research*.

[27] Costa M. A., Wu C. (2006). Tissue engineering of flexor tendons: optimization of tenocyte proliferation using growth factor supplementation. *Tissue Engineering*.

[28] Skopiński P., Zdanowski R., Grzela T. (2012). The influence of sterilized and nonsterilized amniotic dressings on the proliferation of endothelial cells in vitro. *Central-European Journal of Immunology*.

[29] Nasrullah E., Suroto H., Utomo D. N. (2018). *Pengaruh Sterilisasi Radiation Gamma Terhadap Kandungan Growth Factor Pada Sediaan Serbuk Membran Amnion [Effects of Gamma Radiation Sterilization on Growth Factor Level in Amniotic Membrane Powder Preparations]*.

[30] Afify A. E.-M. M. R., Rashed M. M., Mahmoud E. A., El-Beltagi H. S. (2011). Effect of gamma radiation on protein profile, protein fraction and solubility’s of three oil seeds: soybean, peanut and sesame. *Notulae Botanicae Horti Agrobotanici Cluj-Napoca*.

[31] Harrell C. R., Djonov V., Fellabaum C., Volarevic V. (2018). Risks of using sterilization by gamma radiation: the other side of the coin. *International Journal of Medical Sciences*.

[32] Mrázová H., Koller J., Kubišová K., Fujeríková G., Klincová E., Babál P. (2015). Comparison of structural changes in skin and amnion tissue grafts for transplantation induced by gamma and electron beam irradiation for sterilization. *Cell and Tissue Banking*.

[33] Bashandy A. S., Khalaf M. A., Khalaf M. A., AbdElAl M. S., ALGhamry A. A. (2016). Changes in the functional characteristics of amniotic membrane after gamma irradiation. *International Journal of Advanced Research*.

[34] Djefal A., Tahtat D., Nacer Khodja A., Saad Bouzid S., Remane N. (2007). Validation and substantiation of 25 kGy as sterilization dose for lyophilized human amnion membrane. *Cell and Tissue Banking*.

